# Neutrophil Pathways of Inflammation Characterize the Blood Transcriptomic Signature of Patients with Psoriasis and Cardiovascular Disease

**DOI:** 10.3390/ijms221910818

**Published:** 2021-10-06

**Authors:** Amanda Kvist-Hansen, Hannah Kaiser, Xing Wang, Martin Krakauer, Peter Michael Gørtz, Benjamin D. McCauley, Claus Zachariae, Christine Becker, Peter Riis Hansen, Lone Skov

**Affiliations:** 1 Department of Dermatology and Allergy, Copenhagen University Hospital—Herlev and Gentofte, 2900 Hellerup, Denmark; lilian.hannah.kaiser@regionh.fk (H.K.); claus.zachariae@regionh.dk (C.Z.); lone.skov.02@regionh.dk (L.S.); 2 Department of Cardiology, Copenhagen University Hospital—Herlev and Gentofte, 2900 Hellerup, Denmark; peter.riis.hansen@regionh.dk; 3 Department of Medicine, Division of Clinical Immunology, Icahn School of Medicine at Mount Sinai, New York, NY 10029, USA; xing.wang@mssm.edu (X.W.); benjamin.mccauley@mssm.edu (B.D.M.); christine.becker@mssm.edu (C.B.); 4 Department of Clinical Physiology and Nuclear Medicine, Copenhagen University Hospital—Bispebjerg and Frederiksberg, 2400 Copenhagen, Denmark; martin.krakauer@regionh.dk; 5 Department of Clinical Physiology and Nuclear Medicine, Copenhagen University Hospital—Herlev and Gentofte, 2900 Hellerup, Denmark; peter.michael.goertz.02@regionh.dk; 6 Department of Clinical Medicine, University of Copenhagen, 2200 Copenhagen, Denmark; 7 Department of Genetics and Genomic Sciences, Icahn School of Medicine at Mount Sinai, New York, NY 10029, USA

**Keywords:** psoriasis, cardiovascular disease, RNA sequencing, transcriptome, neutrophils, neutrophil to lymphocyte ratio, subclinical atherosclerosis

## Abstract

Background: Patients with psoriasis have an increased risk of atherosclerotic cardiovascular disease (CVD). The molecular mechanisms behind this connection are not fully understood, but the involvement of neutrophils have drawn attention as a shared inflammatory factor. Methods: RNA sequencing using the Illumina platform was performed on blood from 38 patients with moderate to severe psoriasis; approximately half had prior CVD. The neutrophil to lymphocyte ratio (NLR) was obtained from blood samples. Subclinical atherosclerosis was assessed by ^18^F-fluorodeoxyglucose positron emission tomography/computed tomography and ultrasound imaging. Transcriptomic analysis for differential expression and functional enrichment were performed, followed by correlation analyses of differentially expressed genes (DEGs), NLR and subclinical measurers of CVD. Results: 291 genes were differentially expressed between patients with psoriasis with and without CVD. These included 208 upregulated and 83 downregulated DEGs. Neutrophil degranulation was identified as the most significant process related to the upregulated DEGs. Genes for the neutrophil-associated markers MPO, MMP9, LCN2, CEACAM1, CEACAM6 and CEACAM8 were identified as being of special interest and their mRNA levels correlated with NLR, high-sensitive C-reactive protein and markers of subclinical CVD. Conclusions: Patients with psoriasis and CVD had an increased expression of genes related to neutrophil degranulation in their blood transcriptome compared with patients with psoriasis without CVD. NLR may be a potential biomarker of subclinical CVD in psoriasis.

## 1. Introduction

The connection between psoriasis and atherosclerotic cardiovascular disease (CVD) was first discovered and described more than 40 years ago [1]. Patients with psoriasis have an increased risk of CVD and the risk increases with psoriasis severity [2,3,4,5,6]. The molecular mechanisms underlying the connection between these two chronic systemic inflammatory diseases are not fully understood, but the involvement of neutrophils may be an important inflammatory link [7,8,9].

Neutrophils are part of the innate immune system and are the most numerous leukocytes in the blood. They have a short lifespan (1–5 days) and, besides being the first cells recruited to sites of inflammation, these cells act as regulators between the innate and adaptive immune system. The main functions of activated neutrophils include the production of reactive oxygen species, degranulation and the formation of neutrophil extracellular traps. The roles of neutrophils have been described in the immunopathogenesis of both psoriasis and atherosclerosis [10,11,12]. For example, a classic histopathological finding in psoriasis skin is Munro’s micro-abscesses filled with neutrophils [13]. Neutrophils are also a major source of interleukin (IL)-17, and the IL-17 pathway is central to psoriasis pathogenesis [14,15,16]. Moreover, neutrophils are involved in both the early and late stages of atherosclerosis. In the early stages, neutrophil adhesion and extravasation into the arterial wall promote monocyte infiltration and low-density lipoprotein (LDL) cholesterol accumulation in the subintimal layer. In the later stages, neutrophils trigger processes leading to plaque destabilization and, ultimately, atherothrombotic events [10].

The traditional CVD risk factors included in the Framingham risk score underestimate the risk of CVD in patients with psoriasis; therefore, there is a need to find CVD risk factors that are more specific to psoriasis [17,18,19]. The neutrophil to lymphocyte ratio (NLR) is an easily accessible inflammatory biomarker calculated from standard blood tests. NLR is a predictor of CVD and all-cause mortality, and is reduced by targeted anti-inflammatory and lipid-lowering treatment [20]. Both NLR and the total neutrophil count are increased in patients with psoriasis [9,21], and NLR may be a potential biomarker of subclinical CVD in psoriasis [22,23].

The transcriptome of psoriasis skin has been extensively explored and compared with both non-lesional skin and skin from healthy controls [24,25,26], but not much attention has been drawn to the blood transcriptome in psoriasis. Recently, however, blood transcriptomic profiling of patients with psoriasis vs. controls identified inflammasome signaling as the most upregulated canonical pathway in psoriasis and found a common gene transcript signature related to neutrophil-driven inflammation [27,28].

In this study, we explored and compared the blood transcriptome and NLR of patients with psoriasis with and without CVD, and investigated the relationships with non-traditional CVD risk factors and subclinical measures of CVD.

## 2. Results

### 2.1. Baseline Characteristics

Thirty-eight patients with moderate to severe psoriasis were included in the study: 21 with and 17 without CVD (Table 1). Most patients were men (*n* = 28, 73.7%) with a mean age of 60.2 (SD 9.3) years and a mean body mass index of 30.0 (SD 5.4) kg/m^2^. Twenty-four (63.1%) of the patients received systemic anti-psoriatic treatment and 13 (34.2%) had psoriatic arthritis. Ten (26.3%) patients had diabetes, 21 (55.3) had hypertension and 18 (47.4%) received statin treatment. The patients with CVD included eight (38.1%) with myocardial infarction, eight (38.1%) with ischemic stroke, four (19.0%) with peripheral artery disease and one (4.8%) with coronary artery revascularization. Hypertension, diabetes, statin treatment and smoking were all more frequent in patients with CVD. LDL cholesterol levels were lower in the patients with CVD, which reflected the higher frequency of statin treatment in this group. High-sensitive C-reactive protein (hs-CRP) and lymphocyte counts were also lower in the patients with CVD, whereas NLRs were increased in patients with CVD. The data on subclinical measures of CVD were not available for all patients, and measurements included the coronary artery calcium score (CCS; *n* = 25), mean carotid intima–media thickness (CIMT; *n* = 37), and vascular inflammation in the aorta (*n* = 34) and carotid arteries (*n* = 36). All the measures of subclinical CVD were higher in patients with CVD, except for vascular inflammation in the aorta. All baseline characteristics are presented in Table 1.

### 2.2. Differential Expression Analysis Revealed That Processes of Neutrophil Activation Are Upregulated in Patients with Psoriasis and CVD

**Figure 1:**
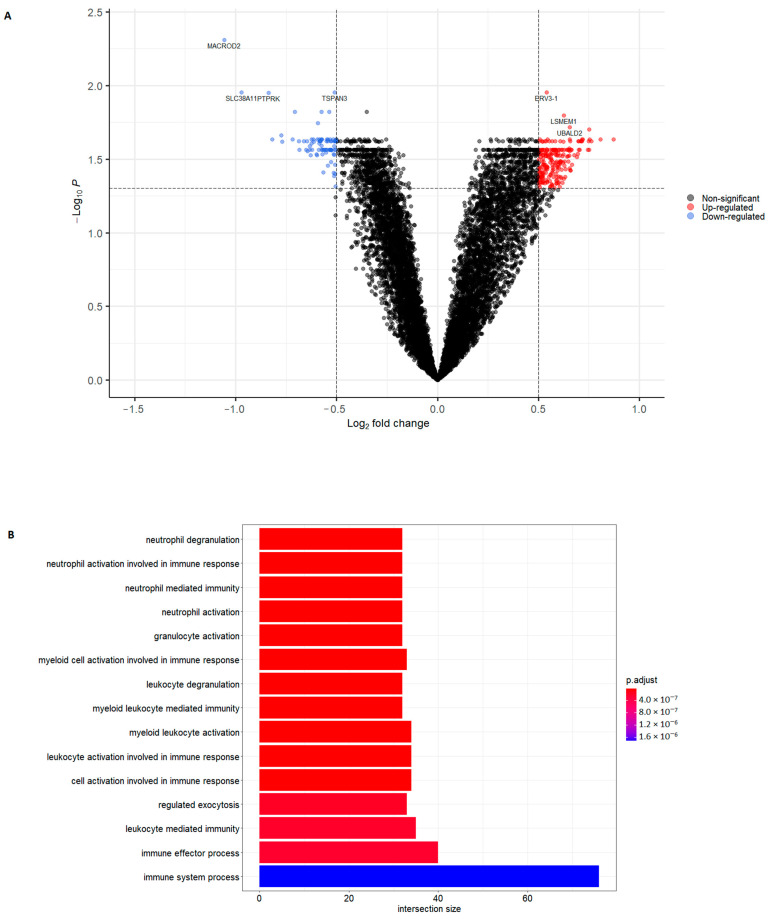
Differential expression analysis comparing patients with psoriasis with and without cardiovascular disease (CVD), and functional enrichment analysis of upregulated differentially expressed genes (DEGs). (**A**) Volcano plot showing the distribution of all protein-coding genes. DEGs were defined by a false discovery rate (FDR) of <0.05 and │log2 fold change│ > 0.50. Dashed lines indicate FDR and log2 fold change thresholds. (**B**) Gene Ontology (GO) functional enrichment for biological processes for the upregulated DEGs. The 15 most significant biological processes are shown. The intersection size indicates how many of the upregulated genes included in the analysis were annotated to the specific GO biological process. Significance level, adjusted *p*-value = 0.05.

Transcriptomic analysis was performed on blood from all patients. We focused on the protein-coding mRNAs and, after filtering, the data contained 10,345 protein-coding mRNAs. Differential expression analysis between patients with and without CVD revealed a total of 291 differentially expressed genes (DEGs), including 208 upregulated and 83 downregulated genes. DEGs were defined by a threshold false discovery rate (FDR) of <0.05 and │log2 fold change│ >0.50. The most significantly upregulated DEGs were *ERV3-1*, *LSMEM1* and *UBALD2*, and the most significantly downregulated DEGs were *MACROD2*, *SLC38A11* and *PTPRK*. The results are presented in Figure 1A and the full list of DEGs is reported in Supplementary Table S1.

Gene ontology (GO) functional enrichment analysis was performed for the biological process GO term on the upregulated and downregulated DEGs separately. The 15 most significant processes that involved the upregulated DEGs are presented in Figure 1B. The most significant process related to the upregulated DEGs was neutrophil degranulation. Further analysis showed that the five most significant results, which were all related to neutrophil processes, were driven by the same 32 upregulated DEGs. For the downregulated DEGs, the most significant process was SRP-dependent co-translational protein targeting the membrane. All significant processes related to the downregulated DEGs are presented in Supplementary Figure S1. Functional enrichment was also performed for the Reactome pathway database (REAC) and Kyoto Encyclopedia of Genes and Genomes (KEGG); the results are reported in Supplementary Figures S2 and S3. For the upregulated DEGs, the most significant pathway identified by REAC was neutrophil degranulation, but no significant pathways were identified by KEGG. For the downregulated DEGs, the most significant pathways identified were eukaryotic translation elongation and ribosome for REAC and KEGG, respectively.

### 2.3. Six DEGs Were Identified as Important Markers of Neutrophil Degranulation

**Figure 2:**
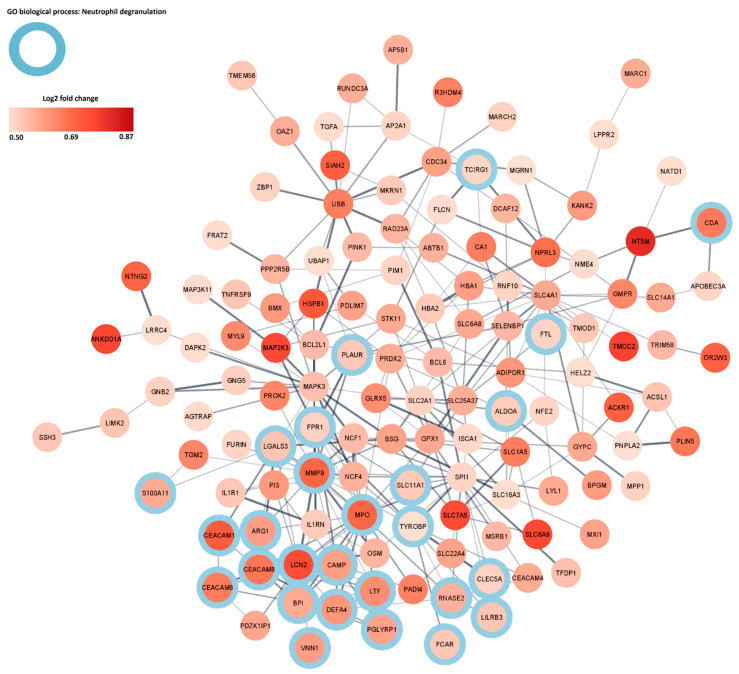
Protein–protein interaction (PPI) network of upregulated DEGs. The network contained 130 nodes and 287 edges. The color gradient of the nodes indicates the log2 fold change of the DEGs, the edge thickness represents the PPI strength, blue circles indicate annotation to GO biological processes of neutrophil degranulation.

To further analyze the importance of the individual DEGs and their relation to neutrophil degranulation, a protein–protein interaction (PPI) network was created from the 208 upregulated DEGs. This resulted in a network containing 130 DEGs and 287 interactions (Figure 2). Twenty-seven of the DEGs contained in the network were related to neutrophil degranulation. A cluster of DEGs related to neutrophil degranulation with a high log2 fold change was identified and included *MMP9*, *MPO*, *LCN2*, *CEACAM1*, *CEACAM6* and *CEACAM8*; these DEGs were selected for further analysis.

### 2.4. Upregulated DEGs Correlated with NLR, CCS, hs-CRP and Vascular Inflammation

**Figure 3:**
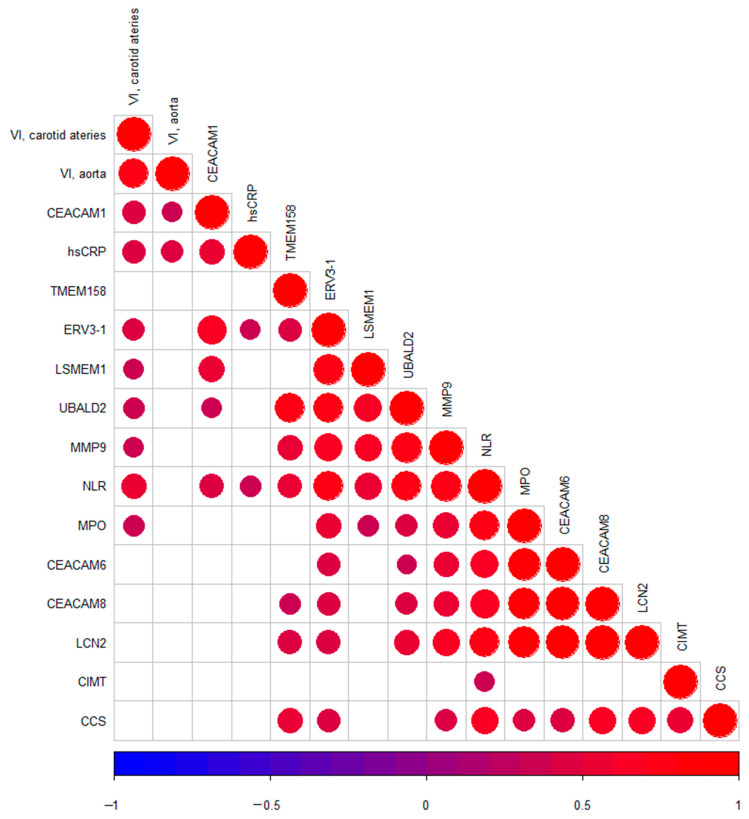
Correlation matrix of DEGs of special interest, non-traditional CVD risk factors and subclinical measures of CVD. Circles indicate a significant correlation (*p* < 0.05). The size of the circles as well as their colors indicates the magnitude of Pearson’s correlation coefficient. NLR (*n* = 38); hs-CRP (*n* = 38); CIMT (*n* = 37); CCS (*n* = 25); VI (carotid arteries, *n* = 36; aorta *n* = 34).

Correlation analyses between non-traditional CVD risk factors (hs-CRP and NLR) and subclinical measures of CVD (CIMT, CCS and vascular inflammation in the aorta and carotid arteries), and upregulated DEGs of special interest were performed (Figure 3). Upregulated DEGs of special interest were selected according to the lowest FDR and highest log2 fold change and according to the results of the PPI network analysis. Upregulated DEGs of special interest included *ERV3-1*, *LSMEM1*, *UBALD2* (these were the most significant DEGs), *TMEM158* (this was the most expressed DEG), *MMP9*, *MPO*, *LCN2*, *CEACAM1, CEACAM6* and *CEACAM8* (these were identified as important in the PPI network analysis). All the selected DEGs correlated significantly with NLR. *ERV3-1* and *CEACAM1* correlated with hs-CPR. *CEACAM1* correlated with vascular inflammation in the aorta, and *CEACAM1*, *MMP9*, *MPO*, *ERV3-1*, *UBALD2* and *LSMEM1* correlated with vascular inflammation in the carotid arteries. *TMEM158*, *ERV3-1*, *MMP9*, *MPO*, *CEACAM6*, *CEACAM8* and *LCN2* all correlated with CCS. None of these DEGs correlated with CIMT.

### 2.5. DEG-Encoded Circulating Proteins Showed a Tendency to Be Increased in Patients with Psoriasis and CVD

**Figure 4:**
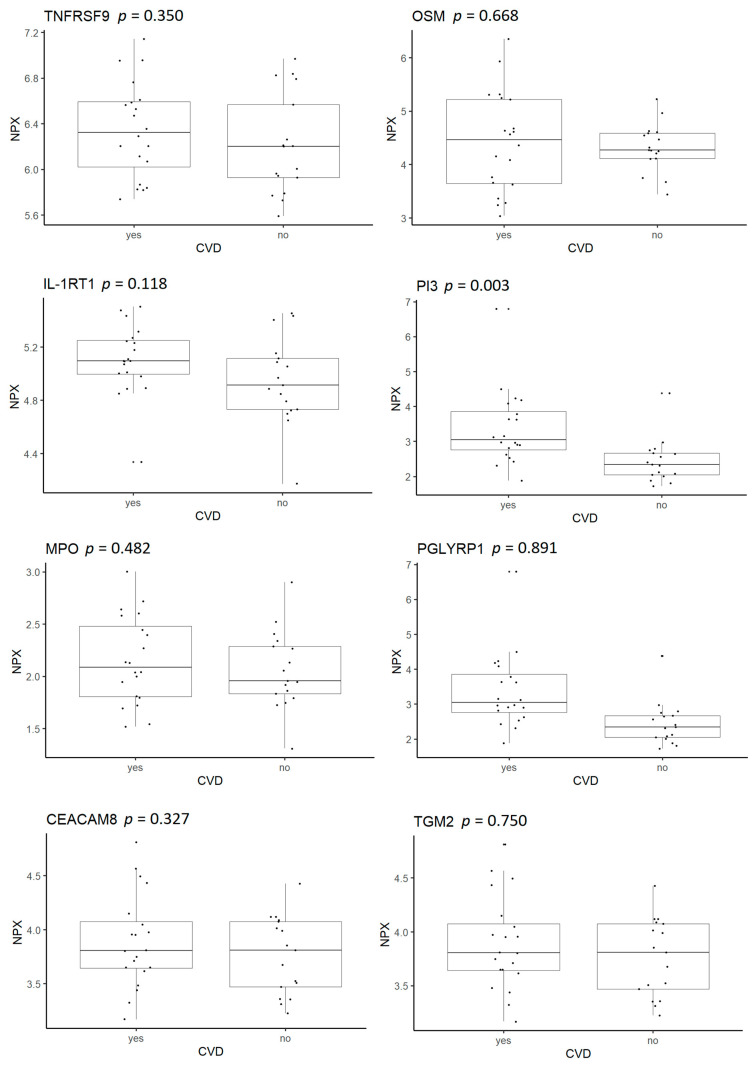
Plasma levels of the proteins TNFRSF9, OSM, IL-1RT1, PI3, MPO, PGLYRP1, CEACAM8 and TGM2 in patients with psoriasis with and without CVD. Boxplots show normalized protein expression (NPX) values of plasma proteins. Significance level, *p* < 0.05.

We investigated if the observed increased expression on the transcriptomic level also resulted in an increased expression on the proteomic level. We queried plasma protein levels in an Olink proteomic dataset that was also available for these patients and contained normalized protein expressions (NPXs) for IL1RT1, MPO, PI3, PGLYRP1, OSM, TGM2, TNFRSF9 and CEACAM8 (Figure 4). NPXs for all these circulating proteins were nominally increased, in agreement with their upregulation at the blood transcriptomic level in the patients with CVD compared with those without CVD, but only the NPX for PI3 was significantly increased (*p* = 0.003).

## 3. Discussion

This blood transcriptomic analysis showed an upregulation of pathways related to neutrophil activation in patients with psoriasis and CVD compared with those without CVD. Indeed, the most upregulated process detected was neutrophil degranulation. We identified six DEGs of special interest, including genes for CEACAM1, CEACAM6, CEACAM8, LCN2, MPO and MMP9, based on a combination of a high log2 fold change and their relation to neutrophil degranulation. All six DEGs of special interest showed a positive correlation to one or more of the examined non-traditional CVD risk factors and subclinical measures of CVD, which reinforced their association with CVD in patients with psoriasis. A positive correlation with NLR was present for all the above mentioned DEGs, in agreement with their relationship to neutrophil degranulation. NLR was increased in patients with CVD compared with those without CVD, and was correlated with hs-CRP, CCS, CIMT and vascular inflammation in the carotid arteries. Other studies have shown a correlation between NLR and non-calcified coronary burden measured by computerized tomography coronary angiography and suggested NLR as a biomarker of subclinical CVD in psoriasis [22,23]. Our results support and extend these findings by showing the correlation of NLR with other subclinical measures of CVD and demonstrating that NLR is also linked to circulating mRNAs and proteins related to neutrophil degranulation.

The blood transcriptome was compared between patients with psoriasis and healthy controls by Rawat et al. [27]. They found that the blood transcriptomic signature of psoriasis was associated with neutrophil-driven inflammation. Interestingly, genes related to neutrophil degranulation were part of this signature. In our study, we also found transcriptomic changes related to neutrophil degranulation in patients with psoriasis, but could further distinguished those as related to patients with psoriasis and CVD compared with those with psoriasis alone.

Among the upregulated DEGs, 32 were related to neutrophil degranulation, which was also the most significant process identified by the GO functional enrichment analysis. Neutrophil degranulation is the process by which granules from activated neutrophils are released. The identified DEGs (*MPO*, *LCN2*, *CEACAM8* and *MMP9*) all encode proteins contained within neutrophil granules [11]. Neutrophil granules are classified into azurophilc granules, specific granules, gelatinase granules and secretory vesicles [11].

During neutrophil degranulation, MPO is released from the azurophilic granules. The actions of MPO include catalyzation of the formation of reactive oxygen species, and increased MPO levels in the blood are associated with conditions characterized by increased oxidative stress and inflammation. Patients with coronary artery disease and peripheral artery disease have increased MPO blood levels, which are associated with poor prognosis and cardiovascular mortality [29]. Notably, in patients with psoriasis, MPO is also increased in both psoriasis skin lesions and in the blood [11,30].

CEACAMs are cell adhesion molecules expressed on epithelial, endothelial and hematopoietic cells with diverse tissue-dependent functions that include tumor suppression, tumor promotion, angiogenesis, regulation of the cell cycle, regulation of adhesion, lymphocyte activation and neutrophil activation. CEACAM1, CEACAM3, CEACAM4, CEACAM6 and CEACAM8 are all expressed on human neutrophils [31]. For example, CEACAM1 is important for angiogenesis and is involved in endothelial homeostasis and dysfunction [32,33], i.e., processes that are part of the pathogenesis of both atherosclerosis and psoriasis [34,35]. In psoriasis lesions, CEACAM1 is expressed in keratinocytes, which are localized at the uppermost layer of the epidermis together with neutrophils, and are considered to contribute to the persistence of neutrophils and inflammation in psoriatic skin [36]. CEACAM6 and CEACAM8 are used as markers of neutrophil activation and specific granules in neutrophils [37]. CEACAM8 is stored in the specific granules and released as a soluble variant upon degranulation [38]. Moreover, CEACAM8-positive neutrophils have been demonstrated in carotid endarterectomy samples from patients with carotid atherosclerosis [39].

LCN2 is a glycoprotein stored within the specific granules of neutrophils that acts in a bacteriostatic manner by binding iron, which is part of a defense mechanism of the innate immune system [40]. LCN2 also acts as an adipokine in adipose tissue, and increased blood levels of LNC2 are associated with metabolic syndrome, and may be a mediator contributing to the low levels of systemic inflammation observed in these patients [41]. LCN2 is increased in the blood and lesional skin of patients with psoriasis, and a correlation with LDL cholesterol levels was also demonstrated in these patients [42,43,44]. Furthermore, LCN2 is considered to promote neutrophil infiltration into the skin, thereby maintaining psoriatic inflammation [11,45]. LCN2 is also increased in the blood of patients with CVD, and studies in rodents have suggested that LCN2 is atherogenic in an enzymatically active complex formed with MMP9 [46]. MMP9 destroys collagen in atherosclerotic plaques, which may cause instability of the fibrous cap and make the plaque prone to rupture and erosion, leading to clinical atherothrombotic events. Indeed, the binding of LCN2 to MMP9 inhibits the degradation of MMP9 and thereby prolongs MMP9 activity [46,47]. Along these lines, interestingly, patients with psoriasis have more coronary atherosclerotic plaques that are prone to rupture compared with healthy controls and demonstrate increased circulating frequencies of the (activated) neutrophil subtype of low-density granulocytes with enhanced capacity for spontaneous neutrophil extracellular trap formation [8,48].

The biology of the other top DEGs according to their FDR value (*ERV3-1*, *LSMEM1* and *UBALD2*) has not been extensively investigated and, to our knowledge, no specific relationships to CVD and psoriasis have been reported.

In the current study, DEG-encoded proteins demonstrated a tendency towards increased levels in the plasma of patients with psoriasis and CVD compared with those without CVD, supporting the results of the transcriptomic analysis and the likely importance of the identified DEGs for CVD pathogenesis in our patients. However, only PI3 plasma levels were significantly increased. PI3 was first discovered in lesional skin from patients with psoriasis [49], is increased in the blood of patients with psoriasis, and correlates to the psoriasis area and severity index (PASI) [50,51].

Study limitations include the lack of control groups, e.g., those without psoriasis or with other systemic inflammatory diseases, which makes it difficult to draw conclusions about whether the findings are limited to patients with psoriasis and CVD. Moreover, the study was based on a relatively small sample size. The study’s strengths include the very extensive and broad clinical examination of the patients. The study population was also homogenous and only included patients with moderate to severe psoriasis.

The results of this study are in line with the emerging evidence that neutrophils play crucial roles in both psoriatic and atherosclerotic inflammation processes and in the interplay between the two diseases. We conclude that patients with psoriasis and CVD have an increased expression of genes related to neutrophil degranulation in their blood transcriptome compared with patients with psoriasis without CVD. Our addition to the existing literature indicates the potential of NLR as a biomarker of subclinical CVD in patients with psoriasis.

## 4. Materials and Methods

### 4.1. Study Population

The study was conducted within a large multi-scale study investigating the connection between psoriasis and CVD (ethical approval No. H-17003458) [52]. Adult patients with a history of moderate to severe plaque psoriasis were recruited from a dermatological hospital clinic in Copenhagen, Denmark. All patients gave informed consent. Moderate to severe psoriasis was defined as a present PASI above 10 or as receiving systemic anti-psoriatic treatment. Approximately half of the patients had CVD, defined as prior myocardial infarction, ischemic stroke, peripheral artery disease and/or coronary artery revascularization more than 6 months before inclusion. All inclusion and exclusion criteria have been described previously [52].

### 4.2. FDG-PET/CT, Coronary Artery Calcium Score and Carotid Artery Ultrasound

The patients were examined by ^18^F-fluorodeoxyglucose positron emission tomography/computed tomography (FDG-PET/CT), and vascular inflammation in the aorta and carotid arteries was determined by FDG uptake. An electrocardiogram-gated low dose CT scan was used to determine CCS; this examination was limited to patients that had not received coronary artery revascularization. Ultrasound imaging was used to determine the CIMT. Calculation of the mean CIMT was taken from measurements of the right and left carotid arteries. The details of these examinations have been described previously [52].

### 4.3. Blood Samples

Blood was collected from all patients and analyzed for lipids, leukocyte counts, hs-CRP and glycated hemoglobin. The NLR was calculated by dividing the total neutrophil count by the total lymphocyte count.

### 4.4. RNA Sequencing

Blood was collected in PAXgene tubes ((BD Bioscience, San Jose, CA, USA) and kept at −20 °C overnight before final storage at −80 °C. The PAXgene Blood RNA kit (QIAGEN, Germantown, MD, USA) was used for isolation of RNA. RNA quality was assessed by the 2100 Bioanalyzer (Agilent, Santa Clara, CA, USA), and sequencing libraries were prepared using TruSeq Stranded Total RNA kits (Illumina, San Diego, CA, USA) for samples with an RNA integrity number of >8. Raw stranded RNA sequencing FASTQ files were quality-trimmed by Trim Galore! (https://www.bioinformatics.babraham.ac.uk/projects/trim_galore/ (accessed on 4 May 2021)). Low-quality bases and reads shorter than 45 bp were removed. Quality review was performed both before and after quality trimming by FastQC (https://www.bioinformatics.babraham.ac.uk/projects/fastqc/ (accessed on 4 May 2021)). Trimmed paired-end reads were aligned to the human hg38 reference genome by the STAR/2.5.4b aligner [53] provided with the splice junction file Homo_sapiens.GRCh38.98.gtf, and duplicates were removed by Picard 2.20.5 MarkDuplicates (https://broadinstitute.github.io/picard/ (accessed on 6 May 2021)). Quantification of the raw counts was performed by Subread 1.6.3 featureCounts and then summarized into a count table.

### 4.5. Targeted Proteomics

Plasma was retrieved and stored at −80 °C. Proteomics analysis was performed using the Olink Proseek Multiplex assay (Olink Bioscience, Uppsala, Sweden). For this study only the plasma concentrations of upregulated DEG-encoded proteins (TNFRSF9, OSM, IL-1RT1, PI3, MPO, PGLYRP1, CEACAM8 and TGM2), which were contained within pre-defined Olink Multiplex panels (Inflammation, Cardiovascular II and Cardiovascular III), were used. Full proteomic data and the method will be reported separately (Kaiser H et al., manuscript in preparation).

### 4.6. Statistical Analyses

All statistical analyses were performed in R version 0.4.5 (R Foundation for Statistically Computing, Vienna, Austria). For descriptive statistics of the baseline characteristics, the mean and standard deviation were used for continuous normally distributed variables, and the median and inter quartile range for continuous non-normally distributed variables. Categorial variables are described as frequencies and percentages. For statistical inferences between groups, Student’s t-test and the Wilcoxon–Mann–Whitney test was used as appropriate for continuous data, and the chi-squared test was used for categorial data.

For transcriptomic data, R package DESeq2 (version 1.30.1) was used for data quality assessment, normalization, filtering and differential expression analysis [54]. The differential expression analysis was performed for the comparison of patients with and without CVD. The analysis was carried out as a multi-factor design accounting for sex, as a separation between men and women was observed during data quality assessments in the sample principle component analysis plot. Within the framework of DESeq2, *p*-values were adjusted for multiple testing by the Benjamini–Hochberg method according to the FDR. DEGs were defined by a combined threshold of FDR < 0.05 and │log2 fold change│ > 0.50. DEGs were visualized in a volcano plot created with the R package EnhancedVolcano (version 1.8.0). GO terms of biological processes, REAC and KEGG were used for functional enrichment [55,56,57,58], which was performed by the gprofiler2 R package (version 0.2.0) [59,60]. The results were adjusted for multiple testing within the default framework of gprofiler2, an adjusted *p*-value of <0.05 was considered to be significant. Functional enrichment was performed for upregulated DEGs and downregulated DEGs separately, and the DEGs were ranked according to their FDR value. The results of the functional enrichment were visualized in bar plots created with the R package enrichplot (version 1.10.2).

The STRING database [61,62,63] was used to create a DEG-encoded PPI network in Cytoscape (version 3.8.2) (https://cytoscape.org/ (accessed on 10 April 2021)). Only the upregulated DEGs were used to construct the PPI network. The log2 fold changes of the DEGs were integrated into the network and presented as the color gradient of the nodes. Edge thickness represents the PPI strength. SRING enrichment was performed with an FDR < 0.05 cutoff. The most significant results of the enrichment are presented in the network as color surrounding the nodes related to the results. Nodes not forming any interaction or only forming a single interaction that was not connected to the large network were removed.

Correlation between DEGs of interest, and NLR, hs-CRP, CIMT, CCS and vascular inflammation in the aorta and carotid arteries were assessed by Pearson’s correlation coefficient within the R package Hmisc (version 4.5-0) and visualized by the R package corrplot (version 0.90). A correlation with a corresponding *p* value of <0.05 was considered significant. DEGs of special interest were selected according to the lowest FDR value and the highest log2 fold change, and from the results of the PPI network analysis.

For proteomics data, the OlinkAnalyze R package (version 1.2.4) (https://github.com/Olink-Proteomics/OlinkRPackage (accessed on 21 February 2021)) was used for data quality assessment and comparisons between groups. Samples that did not pass the quality control and proteins where more than 40% of the samples were below the lower limit of detection were removed. The plasma concentrations of the proteins, provided as the NPX, were compared between patients with and without CVD by a Welch two-sample t-test contained within the OlinkAnalyze package; *p* < 0.05 was considered significant. The results were visualized by box plots created with the R package ggplot2 (version 3.3.5).

## Figures and Tables

**Table 1:** Baseline characteristics of study population.

	Entire Population (*n* = 38)	CVD (*n* = 21)	No CVD (*n* = 17)	*p*-Value
Demographics				
Sex, male	28 (73.7)	17 (80.9)	11 (64.7)	0.258
Age, years	60.2 + 9.3	61.4 + 7.7	58.6 + 10.9	0.374
BMI (kg/m^2^)	30.0 + 5.4	29.2 + 4.9	31.0 + 6.0	0.330
Psoriasis characteristics				
Systemic anti-psoriatic treatment	24 (63.1)	10 (47.6)	14 (82.3)	0.027 *
Psoriasis onset >40 years	10 (26.3)	6 (28.6)	4 (23.5)	0.726
Psoriatic arthritis	13 (34.2)	8 (38.1)	5 (29.4)	0.575
CVD risk factors				
Smoking, current or previous	27 (71.0)	18 (85.7)	9 (52.9)	0.027 *
Diabetes	10 (26.3)	7 (33.3)	3 (17.6)	0.275
Hypertension	21 (55.3)	15 (71.4)	6 (35.3)	0.026 *
Hypercholesterolemia (statin treatment)	18 (47.4)	14 (66.7)	4 (23.5)	0.008 *
HbA1c (mmol/mol)	36.0 (34.0–41.0)	37.0 (35.0–37.0)	36.0 (33.0–37.0)	0.189
Total cholesterol (mmol/L)	4.16 + 1.00	3.65 + 0.85	4.79 + 0.81	0.001 *
LDL-C (mmol/L)	2.18 + 0.76	1.77 + 0.55	2.70 + 0.67	<0.001 *
HDL-C (mmol/L)	1.19 + 0.37	1.18 + 0.39	1.20 + 0.84	0.838
Triglycerides (mmol/L)	1.5 (1.0–2.4)	1.4 (1.0–1.7)	1.7 (1.1–3.7)	0.235
hs-CRP (mg/L)	1.13 (0.70–3.64)	1.01 (0.70–3.82)	1.40 (0.71–2.16)	0.868
Neutrophils (10^9^/L)	3.85 + 1.21	4.33 + 1.24	3.25 + 0.88	0.003 *
Lymphocytes (10^9^/L)	2.05 + 0.78	1.97 + 0.79	2.16 + 0.78	0.470
NLR	2.10 + 0.94	2.44 + 1.00	1.68 + 0.68	0.009 *
Subclinical measures of CVD				
CIMT (mm)	0.73 + 0.13	0.76 + 0.10	0.69 + 0.15	0.153
VI, carotid arteries (TBR_max_)	1.65 + 0.34	1.68 + 0.33	1.56 + 0.30	0.039 *
VI, aorta (TBR_max_)	2.26 + 0.34	2.25 + 0.38	2.27 + 0.31	0.823
CCS	109.0 (1.0–1833.0)	407.0 (283.0–1669.0)	5.0 (0.0–1833.0)	0.005 *

Continuous variables are reported as means ± SDs when normally distributed and as medians (IQRs) otherwise. Categorial variables are reported as frequencies (%). Abbreviations: CVD, atherosclerotic cardiovascular disease; BMI, body mass index; HbA1c, glycated hemoglobin; LDL-C, low-density lipoprotein cholesterol; HDL-C, high-density lipoprotein cholesterol; hs-CRP, high-sensitivity C-reactive protein; NLR, neutrophil to lymphocyte ratio; CIMT, mean carotid intima–media thickness, VI, vascular inflammation; TBR_max_, maximum target to background ratio; CCS, coronary artery calcium score. * Significance.

## Data Availability

The data presented in this study are available on request from the corresponding author.

## Supplementary Materials

The following are available online at https://www.mdpi.com/article/10.3390/ijms221910818/s1. Table S1: List of all DEGs. Figure S1: Functional enrichment of Gene ontology biological processes for downregulated DEGs. Figure S2: Reactome pathway database functional enrichment of up- and downregulated DEGs. Figure S3: Kyoto Encyclopedia of Genes and Genomes functional enrichment of up- and downregulated DEGs.
