# Supplementary material for: Neutrophil Pathways of Inflammation Characterize the Blood Transcriptomic Signature of Patients with Psoriasis and Cardiovascular Disease
Source: Int J Mol Sci. 2021 Oct 6;22(19):10818. doi: 10.3390/ijms221910818 (PMC8509817; doi:10.3390/ijms221910818)

Supplementary material

Figure S1: GO functional enrichment for biological processes of downregulated DEGs. All significant biological processes are reported. Intersection size indicates how many of the down-regulated genes included in the analysis that are annotated to the specific GO biological process. Significance level, p- adjust < 0.05.

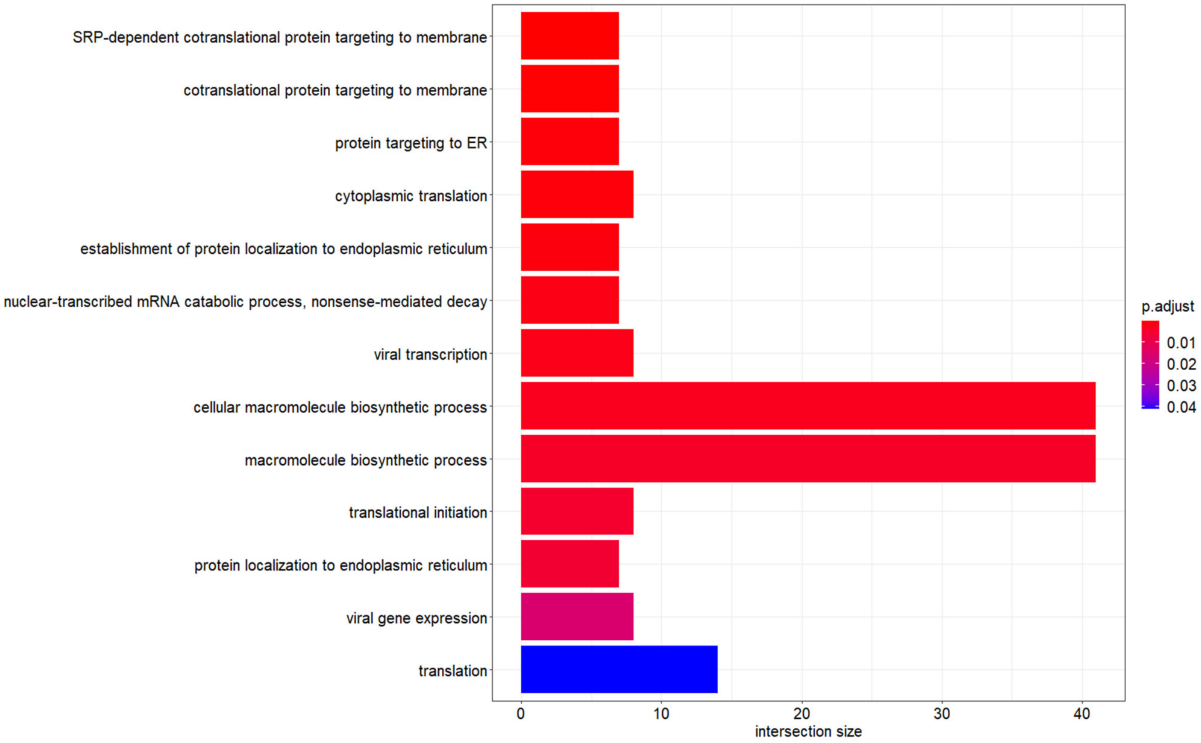

Figure S2: Reactome pathway database functional enrichment of up – and downregulated DEGs. Intersection size indicates how many of the downregulated genes included in the analysis that are annotated to the specific pathway. Significance level, p- adjust < 0.05.

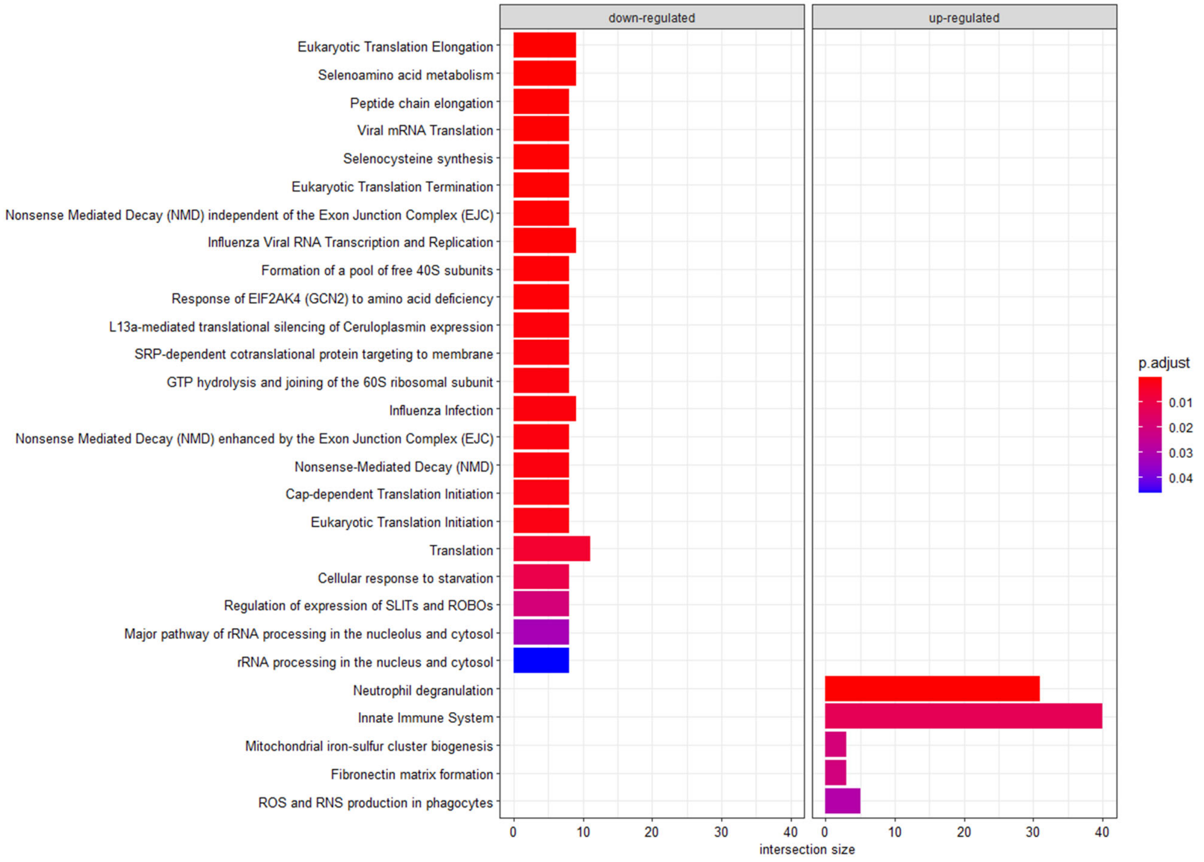

Figure S3: Kyoto Encyclopedia of Genes and Genomes functional enrichment of up- and downregulated DEGs. There were no significant results for the up-regulated DEGs. Intersection size indicates how many of the down-regulated genes included in the analysis that are annotated to the specific pathway. Significance level, p- adjust < 0.05.

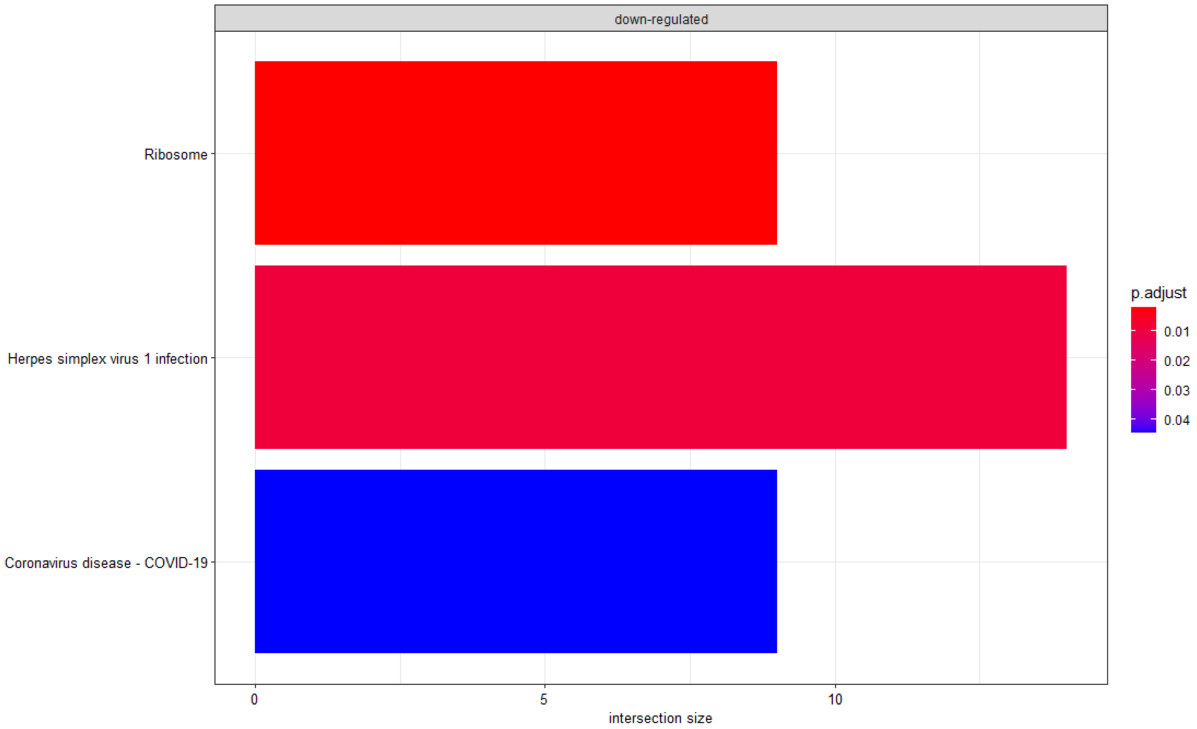

Supplement: Supplementary file 1 [file ijms-22-10818-s001.zip › Supplementary material.pdf]
